# Exploring barriers and facilitators to addressing hazardous alcohol use and AUD in mental health services: a qualitative study among Dutch professionals

**DOI:** 10.1186/s13722-024-00497-z

**Published:** 2024-09-09

**Authors:** Nathalie Kools, Andrea D. Rozema, Fieke A. E. van den Bulck, Rob H. L. M. Bovens, Jolanda J. P. Mathijssen, Dike van de Mheen

**Affiliations:** Tranzo Scientific Center for Care and Wellbeing, Tilburg School of Social and Behavioral Sciences, Tilburg University, PO box 90153, Tilburg, 5000 LE the Netherlands

**Keywords:** Mental health services, Interventions, Hazardous alcohol use, Alcohol use disorder, Qualitative research

## Abstract

**Background:**

Hazardous alcohol use and alcohol use disorder (AUD) are highly prevalent among clients in mental health services, yet significant gaps remain in the adequate assessment of alcohol use and provision of appropriate alcohol interventions. The aim of this study was to conduct an exploration of (i) alcohol intervention elements used in mental health services and (ii) professionals’ reported barriers and facilitators in identifying and intervening with hazardous alcohol use and AUD.

**Methods:**

Qualitative data were obtained by conducting semi-structured interviews among a purposive sample of 18 professionals from 13 different Dutch mental health services organizations (i.e., five integrated mental health organizations with addiction services, five mental health organizations without addiction services, and three addiction services organizations without mental health services). Transcripts were qualitatively analyzed using inductive thematic analysis.

**Results:**

Identified alcohol intervention elements included conducting assessments, brief interventions, treatment, referrals of clients, collaborations with other parties, and providing information to professionals. Professionals mentioned nine barriers and facilitators in the identification and intervention with hazardous alcohol use and AUD, including three aspects of professionals’ behavior (i.e., professionals’ agenda setting, knowledge and skills, and attitudes), actions related to identification and intervening, client contact, collaboration with other parties, and three factors in a wider context (i.e., organizational characteristics, organizational resources, and governmental aspects).

**Conclusions:**

Although diverse alcohol intervention elements are available in Dutch mental health services, it remains unclear to what extent these are routinely implemented. To better address hazardous alcohol use and AUD in mental health services, efforts should focus on enhancing alcohol training, improving collaboration with addiction services, providing appropriate tools, and facilitating support through organizational and governmental measures.

## Introduction

Alcohol use disorder (AUD) and other psychiatric disorders frequently co-occur [1–3]. In fact, people with anxiety or depression disorders are twice as likely to have AUD, and AUD lifetime prevalence among people with severe psychiatric illness ranges between 23.7% and 58.7% [4–7]. AUD co-occurring with other psychiatric disorders (i.e., dual diagnosis) hinders treatment outcomes for psychiatric disorders, exacerbates substance use, increases healthcare utilization, and negatively affects overall quality of life [8–11]. Furthermore, even hazardous alcohol use (i.e., a risky drinking pattern, in which AUD is not [yet] classified) is found to adversely affect clinical course and treatment response in mental health services [12, 13]. Hazardous alcohol use and AUD are highly prevalent among people within mental health services, with rates ranging between 22% and 48% for hazardous use [14, 15] and between 26% and 32% for AUD [16, 17].

Despite this high prevalence within mental health services, alcohol use has been described as ‘the elephant in the room’ (i.e., frequently seen but rarely addressed) [18]. Alcohol use is often poorly assessed, and even when it is assessed adequately, clients are often not provided with appropriate care [19–25]. Appropriate strategies to address alcohol use should, according to World Health Organization (WHO) guidelines, involve screening, brief interventions, and referral to treatment (SBIRT) [26]. This entails the systematic assessment of alcohol use of all clients in mental health services using a validated instrument, like the Alcohol Use Disorder Identification Test (AUDIT), and the provision of brief interventions (typically involving motivational interviewing techniques) when hazardous alcohol use is identified [27]. Additionally, when dual diagnosis is identified, treatment approaches can be categorized as integrated (i.e., both disorders are treated concurrently, by one provider or team knowledgeable in both fields), sequential (i.e., addressing one disorder before the other), or parallel (i.e., treating both disorders simultaneously but by different providers) [28, 29].

SBIRT seems to be less frequently implemented within mental health services than in other care settings, like primary care and general hospitals [18, 22]. Previous studies within mental health services found wide variations in screening methods (e.g., ranging from informal questioning to standardized screening instruments) and screening rates [22, 30–32]. Also, despite promising effects of brief interventions, previous studies reported low rates of applying such interventions to clients with hazardous alcohol use in mental health services [22, 30–32]. To encourage the implementation of identifying and intervening with alcohol use within mental health services, barriers and facilitators that are encountered in this setting should be identified.

Most qualitative studies on hazardous alcohol use and AUD, however, focused on barriers and facilitators of SBIRT implementation in primary care and hospital settings [33–37]. Low SBIRT implementation rates and limited qualitative studies in mental health services might be because promoters and implementers of SBIRT (particularly the WHO) initially developed these programs for primary care and hospital settings, while perhaps overlooking mental health contexts [38]. Thus, similar qualitative studies within mental health services are sparse, with only a few focused on screening and brief interventions [30, 39] or care provision for clients with dual diagnoses specifically [40–43]. For example, across different settings (i.e., primary care and secondary care including hospitals and mental health care organizations), common factors have included knowledge and training for screening and interventions, referral or treatment service availability, and perceived effectiveness of brief interventions [30, 33–37, 39–43]. Additional differences in primary care settings and hospitals include the importance of committed leaders, and grant requirements have seemed more profound, while effective intersectoral communication has appeared especially crucial in mental health services [30, 33–37, 39–43]. Furthermore, mixed findings exist regarding role suitability and attitudes toward working with clients with hazardous alcohol use or AUD in mental health services. For example, some studies indicated that professionals considered it “not my business” and had negative therapeutic attitudes towards working with comorbidity, while other studies showed that professionals did acknowledge their role or had positive attitudes [30, 39–43].

Furthermore, previous studies have highlighted that the operational independence of mental health and addiction services in many Western countries could result in clients with co-occurring disorders receiving insufficient care within one system or facing difficulties in accessing either care system [28, 41, 44, 45]. Additionally, this separation may also hinder the integration of alcohol use as a thematic focus within mental health services. In the Netherlands, specialized mental health services typically focus on specific diagnostic groups and, historically, have operated independently from addiction services, too [44]. In the last decade, about half the Dutch addiction services organizations became part of broader mental health organizations, merging into so-called ‘integrated mental health services organizations’ [46]. However, we note that bringing them together under the same umbrella organization has not necessarily implied integrated treatment provision.

The present study thus added to the literature by eliciting qualitative narratives to examine the identification and intervention of hazardous alcohol use and AUD within mental health services. More specifically, we aimed to conduct an exploration of (i) alcohol intervention elements used in Dutch mental health services and (ii) professionals’ reported barriers and facilitators in identifying and intervening with hazardous alcohol use and AUD.

## Methods

### Study setting

We conducted qualitative, semi-structured interviews with 18 mental health care professionals working in various mental health services organizations throughout the Netherlands. In total, 13 different mental health services organizations were included, including five integrated mental health services organizations (i.e., including both mental health services and addiction services departments), five mental health services organizations (i.e., without addiction services department), and three addiction services organizations (i.e., without a mental health services department). All 13 participating mental health services organizations were nonprofit institutions with agency counseling and were a member of the Dutch sector association for mental healthcare [in Dutch, *de Nederlandse GGZ*], including 107 affiliated organizations in 2021.

### Respondents

Purposive sampling was used for the recruitment of respondents [47]. Our study centered on alcohol interventions within mental health services, including all types of approaches for clients with hazardous alcohol use and AUD, ranging from screenings to collaborations with addiction services. This study involved professionals from both mental health and addiction services to provide a comprehensive perspective on alcohol interventions in the Dutch cascade of care, recognizing addiction services’ integral role in the broader mental health sector. The study focused, however, exclusively on alcohol due to the specific scope defined by the national partnership commissioning the research.

A diverse sample of professional disciplines (e.g., psychologists, psychiatrists, managers, nurse specialists) and organization types were selected through a phased process. First, guidance was sought from members of the national working group “Secondary Care” of the Dutch “Partnership Early Detection of Alcohol”, given their extensive knowledge of alcohol interventions and programs within Dutch mental health services, who suggested various professionals deemed experts in alcohol interventions. Second, some organizations were directly approached using snowball sampling to include a range of disciplines and organization types. The sample selection resulted in a diverse group of professionals with a broad geographical representation across the Netherlands.

In total, 24 professionals were selected and invited for an interview via e-mail, of which 18 participated in the study. Reasons for non-participation were lack of time (*n* = 1), not being employed in the intended department anymore (*n* = 1), not perceiving themselves as a suitable respondent due to lack of working with alcohol interventions (*n =* 1), and non-response (*n* = 3). Among the 18 respondents, 13 were female (72.2%), the average age was 45.6 (*SD* = 9.9) years, the average years of working for an institution was 15.5 (*SD* = 8.7) years, and the average years working in their current position was 8.8 (*SD* = 6.8) years See Table 1.

**Table 1:** Respondent characteristics

	Number of respondents (*n* = 18)	
	*N*	%
**Role**		
Health care psychologist	4	22
Psychiatric nurse practitioner	3	17
Psychiatrist	3	17
Manager	3	17
Director	2	11
(Social) psychiatric nurse	2	11
Prevention worker	1	6
**Organization type**		
MHS organization	6	33
Integrated^a^ MHS organization: MHS department	6	33
AS organization	5	28
Integrated^a^ MHS organization: AS department	1	6

Note. MHS = mental health services. AS = addiction services. ^a^This entails a structure where MHS and AS are part of the same overarching administrative organization; this does not necessarily imply they effectively collaborate or provide integrated treatment together; they may remain distinct in their services

### Procedures

Interviews were conducted in Dutch by the first author (NK, female, junior researcher, MSc.) with previous interview experience. An interview guide was used to conduct the semi-structured interviews, involving two main sections: [1] alcohol intervention elements and [2] barriers and facilitators for identifying and intervening with hazardous alcohol use and AUD. The guide was adapted to respondents’ organization types (e.g., respondents working in integrated organizations were asked about collaborations with addiction services from both within and outside their organization; respondents from addiction services were only asked about their perceptions on collaborations for clients with hazardous alcohol use or AUD within mental health services). Examples of interview questions for professionals in mental health services (departments) were: *“Do professionals at your organization ask about their clients’ alcohol consumption during intakes? And in what way does this happen?”* and *“What facilitates/hinders the collaboration with addiction services (departments) for clients identified with AUD within the mental health institution?”*

All interviews were conducted via video-calls and were audio-recorded. No other people were present at any interview beyond the individual respondent(s) and interviewer. Two respondents were interviewed in a duo-interview. Interviews took on average 60 min (*SD* = 8 min) and were conducted between February and May 2021. Interviews were transcribed verbatim by professional transcriptionists. No major new topics came up in the last few interviews, indicating saturation. The Consolidated Criteria for Reporting Qualitative Research (COREQ) checklist [48] guided the reporting of methods and results in the present study.

### Analysis

Transcripts were qualitatively analyzed in Dutch using inductive thematic analysis, in which coding and theme developments were driven by the data and reflected the explicit content of the data [49]. Transcripts were first coded by the third author (FvdB) in the software package ATLAS-Ti 8 [50], distinguishing between the two research questions by the classifications ‘alcohol intervention element’, and ‘barrier and facilitator’. In addition, 30% of the transcripts were coded independently by the first author (NK) and then compared and discussed until agreement was reached, as coding can be seen as flexible and organic and should evolve throughout the coding process [49]. Code groups were then created, which were classified into general themes. Subsequently, the first author (NK) conducted substantial iterative revisions on the code groups and themes during intensive consultations with the second author (ADR) until consensus was reached about the code groups and overarching general themes. These were translated into English. Finally, in consultation with all the co-authors, the appropriateness of the developed themes and code groups were discussed and adjusted as necessary.

## Results

Study results are categorized into two sections: (1) identified alcohol intervention elements and (2) barriers and facilitators for identifying and intervening with hazardous alcohol use and AUD.

### Identified alcohol intervention elements

Several alcohol intervention elements were identified and divided into six categories: alcohol assessment, brief intervention, treatment, referral, collaborations, and information provision. These categories, related elements, and further explanations or examples are presented in Table 2.

**Table 2:** Identified alcohol intervention elements

Category	Alcohol intervention element	Explanations or examples
1. Assessment	1.1 With simple, standard questions	N/A
	1.2 With validated screening instrument	e.g., Alcohol Use Disorder Identification Test-Consumption (AUDIT-C); Measurements in the Addictions for Triage and Evaluation (MATE)
2. Brief intervention	2.1 Psychoeducation	N/A
	2.2 Motivational interviewing	Including advice on alcohol intake or seeking treatment (e.g., detoxing or apply to addiction services)
3. Treatment	3.1 Integrated treatment within mental health services	Treating alcohol use and other mental health problems by one provider or team
	3.2 In collaboration with addiction services (parallel treatment)	Treating alcohol use and mental health problems simultaneously by different providers, e.g., collaborating with addiction services during intake phase to set up a plan for simultaneous treatment or referring to addiction services while continuing treatment at mental health services
4. Referral	4.1 To addiction services (sequential treatment)	First addressing alcohol use before returning to mental health treatment
	4.2 To in-house alcohol clinic (parallel treatment)	N/A
	4.3 To dual diagnosis facility (integrated treatment)	N/A
	4.4 Back to general practitioner	If hazardous alcohol use or AUD led to rejection from mental health services
5. Collaborations	***With addiction services***	
	5.1 Consultations	Including consultations, advice, structural deployment of addiction professionals within mental health services, and regular meetings to review cases and make agreements
	5.2 (In)formal collaborative agreements	Collaboration aspects (e.g., consultations or referral practices) formalized through agreements or established informally through practical, on-the-ground arrangements
	***With other external parties***	
	5.4 With social domain	e.g., home care in case of simultaneous need for physical care, community welfare organizations, or assisted living facilities
	5.5 With primary care mental health worker	N/A
6. Information provision	6.1 Training professionals	e.g., e-learning or training days in alcohol assessment, dual diagnosis treatment, and motivational interviewing
	6.2 Protocols	e.g., for screening instruments and integrated treatment methods

### Barriers and facilitators

Several barriers and facilitators for the identification of and interventions for hazardous alcohol use and AUD were identified (Table 3). These factors mainly consisted of two sides of the same coin: the barrier (when absent) and facilitator (when present) sides.

**Table 3:** Professionals’ reported barriers and facilitators

Barriers		Facilitators	
Factor	Code	Factor	Code
1. Limited agenda setting among professionals	1.1 Prioritizing other themes 1.2 Underestimating necessity 1.3 Perceived as extra work 1.4 Lack of personal affinity 1.5 Losing key persons	1. Agenda setting among professionals	1.1 Project champion(s) 1.2 Recognizing necessity 1.3 Motivation through personal affinity 1.4 Continuity in attention 1.5 Creating support 1.6 Agenda setting at general practitioners
2. Lack of knowledge and skills	2.1 Lack of knowledge 2.2 Professional hesitation 2.3 Rapid knowledge erosion 2.4 Limited training curriculum	2. Sufficient knowledge and skills	2.1 Having knowledge 2.2 Comprehensive training curriculum 2.3 Ongoing education on-the-job 2.4 Knowledge exchange between mental health and addiction services
3. Hindrance in professional attitude	3.1 Alcohol-related stigma and taboos 3.2 Lacking integrated treatment vision	3. Supportive professional attitude	3.1 Reducing alcohol-related stigma and taboos 3.2 Integrated treatment vision 3.3 Providing tailored care
4. Lack of action	4.1 Lacking alcohol assessment 4.2 Lacking follow-up actions after identification	4. Supportive actions	4.1 Adequate alcohol assessment 4.2 Using screening instrument(s) 4.3 Incorporating theme into mental health treatment 4.4 Involving “support system” of client during intake/treatment
5. Difficulties in client contact	5.1 Client resistance 5.2 Contact loss due to referral errors	5. Good client contact	5.1 Establishing good therapeutic relationship
6. Difficult collaboration	***With addiction services*** 6.1 Poor contact and communication 6.2 Inadequate client referrals 6.3 Lack of awareness regarding available services 6.4 Differences in treatment approaches and vision 6.5 Overconfidence in own approach 6.6 Resistance to change 6.7 Disagreements over roles 6.8 Financial self-interest 6.9 Lack of shared responsibility 6.10 Bureaucracy 6.11 Inability to access each other’s electronic health records	6. Effective collaboration	***With addiction services*** 6.1 Close contact and communication 6.2 Awareness of available services 6.3 Acceptance of each other’s expertise 6.4 Consultations 6.5 Seamless and coordinated client referrals 6.6 Integrated collaboration 6.7 Shared commitment 6.8 Joint evaluation of collaboration 6.9 Willingness to experiment 6.10 Establishing collaborative agreements ***With other parties*** 6.11 Collaboration with social domain 6.12 Collaborative network with other healthcare organizations
7. Limiting organizational characteristics	7.1 Lack of appropriate treatments 7.2 Mental health care silos 7.3 Large, cumbersome organizations 7.4 Insufficient management support	7. Organizational characteristics	7.1 Management endorsement 7.2 Involving experts 7.3 Having an alcohol clinic 7.4 Having a dual diagnosis department 7.5 Offering integrated treatment
8. Limited organizational resources	8.1 Time constraints 8.2 Insufficient staffing 8.3 Funding constraints 8.4 Lengthy waiting lists		
9. Governmental barriers	9.1 Inadequate health insurance reimbursement 9.2 Lack of alcohol theme in clinical guidelines	9. Governmental support	9.1 Incorporated in treatment guidelines 9.2 Legislation 9.3 Adequate health insurance reimbursement 9.4 Government campaigns for alcohol prevention

#### Agenda setting

Respondents mentioned that professionals often prioritized other themes over addressing alcohol use, perceiving it as too much extra work and underestimating its importance. ‘Project champions’, or professionals with affinity for the theme, were seen as vital facilitators in the implementation of alcohol-related initiatives by feeling responsible for the theme and serving as primary points of contact. However, the focus on alcohol was therefore often dependent on these key people, meaning that if they left, this focus could disappear from the agenda. Continuous reminders (e.g., integrating the theme in meetings and sharing information through intranet or presentations) and creating team support for new alcohol-related methods were deemed important for maintaining continuity. Finally, general practitioners were encouraged to assess their patients for alcohol use before referring them to mental health services.

“Everyone is so busy with their own patients and what they all have to do, so very often things are forgotten. So, you have to … if, when they see me walking down the corridor, they are all thinking about alcohol” – #9, psychiatrist, integrated mental health services (mental health services department).

#### Knowledge and skills

Respondents noted professionals’ lack of knowledge and hesitation to act regarding substance use. They emphasized the need for comprehensive training in general knowledge about substance use, identification, and intervention methods (e.g., applying screening instruments and motivational interviewing), and in-house treatment methods. To convey this knowledge effectively, comprehensive training during professional education, ongoing education on-the-job, and knowledge exchange between mental health and addiction services were highlighted as effective methods.

#### Professional attitude

Stigmatization was noted as a barrier, with individuals with hazardous alcohol use or AUD often viewed as more responsible for their problems compared to other psychiatric disorders. Additionally, alcohol was called a taboo subject, making professionals hesitant to bring it up for fear of being overly intrusive.

“I think that people, including treatment providers, often see alcohol as someone’s own responsibility, and they choose it for themselves. And there is much more empathy and sympathy for people, for example, with depression or an anxiety disorder. While addiction problems are, of course, also a psychiatric issue” – #4, health care psychologist, integrated mental health services (mental health services department).

According to respondents, there was also a lack of an integrated treatment vision among many professionals due to a perceived distinction between addiction and psychiatry, which often resulted in alcohol use being ignored (i.e., “not our business”) or referrals to addiction services to “treat the addiction first”. Reduced stigma and more tailored care supported by integrated treatment vision were suggested as solutions.

“I often notice now that people are referred to us, and mental health care lets them go, whereas I think ‘Please don’t do that’, because there is also a psychiatric problem for which they have the expertise. But mental health care quickly tends to, that’s my impression, to place people with us to treat the addiction first” – #3, health care psychologist, addiction services.

#### Actions related to identification and intervening

Respondents mentioned inadequate assessment of clients’ alcohol use often due to insufficient probing, other priorities, or simply forgetting it. Additionally, respondents noted that identified hazardous alcohol use often lacked follow-up actions. Respondents emphasized the importance of thorough and routine screening, supported by user-friendly screening instruments (e.g., AUDIT, MATE, Routine Outcome Monitoring [ROM], and alcohol breath tests).

“It might also be something which you shouldn’t just ask during intake but, for example, should ask again during an evaluation process because I can also imagine that at the start of treatment, people don’t always talk about it. And when you treat them for a different complaint, you ask again, and you might discover it then” – #4, health care psychologist, integrated mental health services (mental health services department).

Other suggested strategies were stricter triaging processes, for example by including standard consultations with addiction experts as part of the intake or treatment process. Respondents also advocated for using motivational interviewing and psychoeducation during the assessment phase to increase awareness about alcohol-related issues. Finally, integrating alcohol more structurally into treatment plans and involving clients’ support systems were noted as beneficial strategies.

#### Client contact

Noted barriers were expectations of resistance from clients (e.g., such as in disclosing alcohol use, underestimating the problem, or resisting certain treatments) and referral errors that often resulted in loss of contact with clients already difficult to retain. Building trust through a strong therapeutic relationship was seen as a way to improve client engagement and retention.

“I prefer to do that at a moment when someone has some trust in me, then I find it okay. You also have to assess the willingness of a patient during the intake, because if you focus too much on alcohol, they may sometimes stay away, for example” – #9, psychiatrist, integrated mental health services (mental health services department).

#### Collaborations

Collaborations were categorized into collaborations between mental health and addiction services versus collaborations between mental health services and other parties. Regarding collaborations between mental health and addiction services were barriers related to poor contact and communication. Inadequate or mismatched client referrals were often compounded by a lack of awareness of available services amid a rapidly changing “social map”. Respondents mentioned the importance of knowing each other personally, regular and direct contact via contact persons and site visits, awareness of available services, mutual expectations, and respect for each other’s expertise.

“Sometimes we have trouble reaching the consulting doctor. Someone else steps in, although there are agreements about it. So, we have to repeat those appointments again and then it’s okay” – #15, social psychiatric nurse, mental health services.

“Get to know each other and involve each other early on. Join each other’s multidisciplinary meetings. Uhm, just pick up the phone sometimes, even when nothing is going on, not always just, um, throwing complicated casuistry over the fence at each other, but just working together” – #7, psychiatrist, addiction services.

Respondents also expressed attitudinal obstacles, including differences in treatment approaches (i.e., individual-focused, disease-oriented mental health services versus systemic, societal-oriented addiction services), which hindered collaboration due to entrenched views and resistance to change. Respondents noted disagreements over roles and shared responsibility, with professionals arguing that clients should be treated by the other party while avoiding other clients due to financial self-interest. Boundary conditions, such as bureaucratic constraints and difficulties accessing electronic health records, were raised as other barriers.

Conversely, actively involving each other in treatment processes were highlighted as facilitators of collaboration (e.g., consulting each other, seamless and coordinated referrals, and a shared commitment to offer adequate treatment in a coordinated, integrated manner). Joint evaluations, experimentation with innovative solutions, and collaborative agreements were seen as enhancing collaboration effectiveness.

“And if you have a complicated issue, don’t shy away from it, don’t fling things at each other over the fence, but find solutions together. And that’s all, yes, almost continually keeping up a kind of collaborative morale” – #7, psychiatrist, addiction services.

Regarding collaborations with other parties, respondents emphasized the necessity of working in a collaborative network with other healthcare organizations to enhance care quality, such as organizing meetings with the social domain (e.g., social work) more frequently.

“You do see that the organizations are coming closer together, that there’s more overlap. And they’re increasingly seeking collaboration (…). So, within the organizations, you actually see that they try to broaden knowledge in certain areas. And you see, because ultimately that’s not the only solution. That collaboration is also increasing. So, those network structures are actually only growing because people increasingly see that you can’t do it alone” – #12, director, addiction services.

#### Organizational characteristics

Respondents described a lack of facilities or services for clients with both alcohol and other psychiatric problems who did not “fit” in neither mental health services nor addiction services (e.g., clients with psychosis sensitivity or intellectual disability), hindered by overspecialization in separate “silos” and bureaucratic challenges in large organizations.

“We still have people of whom we think: ‘Where should they go? Where do they belong?’ So, you can say there is a gap in the market. We’ve created something, but that also creates new gaps in the market” – #3, health care psychologist, addiction services.

Supportive managers who facilitate or even put pressure on implementing alcohol-related interventions and related collaborations was seen as crucial. Additionally, addiction expertise within mental health services and integrated treatment structures, whether offered internally or through referrals to specialized care (i.e., alcohol clinics and dual diagnosis departments), were considered “facilitating”.

“Any department at [MHC] that has to collaborate with another department runs into the question of ‘How are we going to do that?’ Are we going to open separate client records, are we going to do one client records, who is going to be chief practitioner, what does that mean, what are your responsibilities then? That’s what people are running into everywhere. And I think that organizations should take a bit more, um, of a lead in that and just start saying ‘Gosh, hey, this is how we’re going to do it, and this is what it means for the [chief practitioner], this is what it means for client record formation’” – #5, health care psychologist, mental health services.

#### Limited organizational resources

Respondents cited time constraints, insufficient staffing, and funding issues as major barriers. High work pressure contributed to inadequate assessment and follow-up of clients’ alcohol use to not assessing clients’ alcohol use (adequately) or following up after identification. Respondents noted that funding constraints (e.g., budget cuts, narrow profit margins, reliance on external funding of insurers) exacerbated these barriers. Additionally, lengthy waiting lists led to accelerated rejections of clients with hazardous alcohol use or AUD who may otherwise have benefited from treatment within mental health services.

#### Governmental aspects

Respondents mentioned that health insurance reimbursements were a significant barrier, as certain mental health services could not work with AUD diagnoses due to a lack of contractual agreements with insurers. Treating clients in parallel or integrated with addiction services was also described as difficult because of reimbursement rules. Additionally, respondents mentioned that alcohol is often not explicitly included in clinical guidelines for mental disorders, undermining its inclusion in daily practice. Better integration in daily clinical practice could, according to respondents, be achieved through improved guidelines and legislation promoting alcohol-related working methods (i.e., screening or seeking collaborations). This could be further supported by broadening health insurance reimbursements, for example through incentive fundings for new initiatives and prevention activities. Finally, enhanced awareness of alcohol prevention through governmental campaigns was mentioned to be beneficial, too.

“It was going well for quite some time until the health insurer really said that addiction care can only be provided by addiction services, and other psychiatric issues can only be treated by general mental health care. (…) We can hardly provide integrated treatment, because then you have double patient records open, and that becomes way too expensive. Well, then you get that kind of issue. So instead of making healthcare cost-effective, it becomes very complex and expensive” – #1, psychiatric nurse practitioner, integrated mental health services (addiction services department).

## Discussion

### Key findings

This study aimed to explore alcohol intervention elements used in Dutch mental health services and professionals’ reported barriers and facilitators in identifying and intervening with hazardous alcohol use and AUD. Identified alcohol intervention elements were diverse and included conducting assessments, brief interventions, treatment within the organization, referrals of clients, and collaborations with other parties (i.e., addiction services and social domain). Additionally, professionals were supported through training or protocols. Furthermore, professionals mentioned nine barriers and facilitators in the identification and intervention with hazardous alcohol use and AUD, including aspects of professionals’ behavior (i.e., professionals’ agenda-setting, knowledge and skills, attitudes), actions related to identification and intervening, client contact, collaboration with other parties, and factors in a wider context (i.e., organizational characteristics, organizational resources, and governmental aspects).

### Interpretation of key findings

This study identified various alcohol intervention elements that, combined, closely resembled SBIRT approaches [51]. However, there was significant diversity within each intervention category, like different screening methods. International SBIRT research in mental health services also found wide variations in screening methods and identified diverse rates of screening, along with low rates of brief interventions [22, 30, 31]. These findings raise questions about the consistency and implementation levels of alcohol interventions in Dutch mental health services, which future quantitative research should verify. If similar trends are observed in Dutch services, efforts should prioritize the consistent integration of SBIRT elements to improve screening and intervention rates and ultimately ensure effective management of hazardous alcohol use and AUD.

Professionals’ knowledge, skills, and attitudes were reportedly crucial, consistent with prior research [30, 31]. Enhancing these competencies through alcohol training appeared prerequisite to the implementation of alcohol interventions within mental health services, as it increases professionals’ engagement in alcohol-related tasks (e.g., SBIRT and initiating treatment themselves) and improves alcohol-related knowledge, skills, and attitudes towards working with people with hazardous alcohol use and AUD [31, 48]. The beneficial influence of training on professional attitudes has seemed important, since negative attitudes of professionals towards patients with hazardous alcohol use and AUD were found to contribute to suboptimal health care [52]. We also identified attitudinal barriers (i.e., alcohol-related stigma and taboo and lack of integrated treatment visions) that may be addressed through regular training programs involving addiction services professionals and people with lived experience [52, 53]. Yet, since previous research in primary health care settings found no associations between professionals’ attitude changes and increased rates of screening and brief interventions [54], these findings underscore the need to further study attitudinal effects on identifying and intervening in mental health services settings.

Furthermore, although dual diagnosis was not our study’s primary focus, the ‘referral to treatment’ element of SBIRT approaches identified in this study needs further clarification. Referral to treatment in other settings, like primary care and hospitals, often entails referring clients with (suspected) AUD to addiction services. However, this might be inappropriate for clients in mental health service contexts. Research recommends integrated approaches for dual diagnosis over sequential and parallel approaches due to various drawbacks of the latter two [28, 55]. Referrals for clients with a dual diagnosis should therefore ideally lead to integrated treatment. However, all three treatment approaches (i.e., integrated, sequential, and parallel) were mentioned by our respondents, indicating that referrals without integrated treatment were still common practice.

Indeed, many drawbacks associated with sequential and parallel approaches aligned with barriers to collaboration were identified in this study, including poor contact between services, differences in treatment approaches, challenges in accessing or integrating administrative systems, and a lack of shared responsibility [28, 29, 44, 45, 56]. Implementing integrated approaches is complex, however, involving extensive additional training and supervision to deliver integrated treatment and financial implications of making related organizational changes [57]. Aligning with prior systematic review findings, respondents in the present study expressed a compelling need for increased collaboration between mental health and addiction services with a joint willingness to explore new coordinated intervention models [58].

This study included professionals from three organization types: integrated mental health services, mental health services, and addiction services. Previous research found no difference in the nature and numbers of addiction treatments between integrated and non-integrated services, suggesting that organizational integration did not necessarily improve access to addiction services [46]. However, these findings relate to AUD treatment rates only, leaving it unclear about possible differences between organization types in addressing hazardous alcohol use. Therefore, future research might determine whether integration improves the identification and intervention of hazardous alcohol use or the provision of integrated treatment for dual diagnosis within mental health services.

This study’s limitations should impact the interpretation of its findings, however. First, findings may not universally apply to countries with different mental health and addiction service structures, like fully integrated structures. Findings are likely most relevant to systems that have separate services for mental health and addiction care. Second, our purposive sampling strategy may have resulted in a selection bias among respondents: Those who were chosen and/or willing to participate might have had more experience or knowledge about alcohol use in mental health services than the average professional, and this might have led to more favorable attitudes, meaning that the actual state of identification and intervention within the Netherlands might be even less positive.

Nevertheless, the present study included a heterogenous group of mental health care professionals from various organization types, which resulted in a broad exploration and rich dataset on a complex topic for improving mental health care for clients with hazardous alcohol use and AUD. Furthermore, the study’s findings highlight the importance of developing policies in Dutch mental health services that address the identified barriers by enhancing alcohol training, improving collaboration with addiction services, providing appropriate tools, and facilitating support through organizational and governmental measures. This might facilitate the integration of comprehensive alcohol interventions, leading to the better identification and management of hazardous alcohol use and AUD within mental health services.

## Conclusions

Although diverse alcohol intervention elements are available in Dutch mental health organizations, it remained unclear to what extent these are routinely implemented. To better address hazardous alcohol use and AUD, stakeholders should focus on enhancing alcohol training, improving collaboration with addiction services, providing appropriate tools, and facilitating support through organizational and governmental measures.

## Data Availability

The data generated and analyzed during this study are not publicly available due to ERB restrictions. Non-identifiable data are, however, available from the authors upon reasonable request and with permission from the ERB of Tilburg University.

## Abbreviations

AUD: Alcohol use disorder
SBIRT: Screening, brief intervention, and referral to treatment
AUDIT: Alcohol Use Disorder Identification Test
AUDIT-C: Alcohol Use Disorder Identification Test-Consumption
MATE: Measurements in the Addictions for Triage and Evaluation
ROM: Routine Outcome Monitoring
